# Global research trends in tumor stem cell-derived exosomes and tumor microenvironment: visualization biology analysis

**DOI:** 10.1007/s00432-023-05450-2

**Published:** 2023-11-02

**Authors:** Ziwei Guo, Gang Wang, Zhangjun Yun, Yanbo Li, Bohao Huang, Qian Jin, Yue Chen, Luchun Xu, Wenliang Lv

**Affiliations:** 1 Department of Infection, China Academy of Chinese Medical Sciences, Guang’ anmen Hospital, Beijing, China; 2 Dongzhimen Hospital, Beijing University of Chinese Medicine, Beijing, China; 3 Guang’ anmen Hospital, Beijing University of Chinese Medicine, Beijing, China

**Keywords:** Cell-derived exosomes, Tumor microenvironment, Bibliometric analyses, Visualization, CiteSpace, VOSviewer

## Abstract

**Bankground:**

The tumor microenvironment (TME) is an internal environment composed of various cells and an extracellular matrix. Cancer stem cell-derived exosomes (CSC-Exos), as essential messengers involved in various tumor processes, are important carriers for bidirectional communication between the tumor microenvironment and tumor cells and play an important role in the tumor microenvironment. Nevertheless, few bibliometric analyses have been systematically studied in this field.

**Methods:**

Therefore, we aimed to visualize the research hotspots and trends in this field through bibliometrics to comprehend the future evolution of fundamental and clinical research, as well as to offer insightful information and fresh viewpoints. The Scopus database was used to search the research literature related to exosomes and tumor microenvironments after the establishment of this repository. CiteSpace (version 5.8.R3) and VOSviewer (version 1.6.16) were used for visualization and analysis.

**Results:**

In this study, a total of 2077 articles and reviews were included, with the number of articles on exosomes and tumor microenvironments significantly increasing yearly. Recent trends showed that the potential value of exosomes as “tumor diagnostics” and “the application prospect of exosomes as therapeutic agents and drug delivery carriers” will receive more attention in the future.

**Conclusions:**

We revealed the current status and hotspots of tumor stem cell-derived exosomes and tumor microenvironments globally through bibliometrics. The prospect of the regulatory role of CSC-Exos in TME, the potential value of diagnosis, and the application of drug delivery vectors will all remain cutting-edge research areas in the field of tumor therapy. Meanwhile, this study provided a functional literature analysis for related researchers.

## Introduction

Exosomes are bilayered membranous vesicles 30–100 nm in diameter that originate from the endocytic pathway in most cells and are released from the multivesicular body (MVB) into virtually all biological fluids such as blood, saliva and urine (Colombo et al. 2014a; Kalluri and LeBleu 2020). In 1983, Johnstone (Pan et al. 1985) isolated a small vesicle from the supernatant of sheep reticulocytes, which was initially thought to be a cell-secreted waste, and later, with the study of exosomes, these vesicles were found to be enriched with a variety of components, such as cell-specific proteins, lipids, mRNA, microRNA (miRNA) and other non-coding RNAs (Tomasetti et al. 2017; Pegtel and Gould 2019). Exosomes, secreted by a variety of cells, such as tumor cells, macrophages, fibroblasts, etc., are widely distributed in various body fluids, such as blood, urine, peritoneal fluid, synovial fluid, milk, etc. (Kalluri and LeBleu 2020; Zhang et al. 2020; Caby et al. 2005), which act on target cells by carrying and delivering key signaling molecules, thus affecting the physiological and pathological state of the target cells (Camussi et al. 2010; Zhang and Yu 1871; Zhang et al. 2018).

The tumor microenvironment, a stable local environment composed of tumor cells, macrophages, fibroblasts, and extracellular matrix, plays a vital role in the occurrence, recurrence, metastasis, and chemotherapy resistance of cancer (Ribatti and Vacca 2008; Mantovani et al. 2008). An increasing number of studies have shown that exosomes play an important role as carriers in mediating cellular communication and material exchange between CSCs and tumor cells as well as other cells in the microenvironment and regulate the processes of tumor growth and metastasis, drug resistance, angiogenesis, and immune escape by transporting tumor-related mRNAs, miRNAs, proteins, etc. (Cho et al. 2012; Bobrie et al. 2012; Peitzsch et al. 2017). In addition, exosome-carrying molecules are considered to be important markers for tumor diagnosis and prognosis (Liu et al. 2022a). A study found that the molecular composition of these malignant melanoma vesicles produced by CSC differs from that of differentiated tumor cells (Palacios-Ferrer et al. 2021). These molecules exhibit variability in patients with malignant melanoma compared to healthy individuals. It has also been found that circCARM1, secreted by breast cancer stem cells (BCSCs), is crucial in breast cancer cell glycolysis through the miR-1252-5p/PFKFB2 signaling axis (Liu et al. 2022b). These studies have demonstrated that cancer stem cell-derived exosomes are becoming a hot research topic in tumor microenvironments due to their properties and functional attributes.

Exosomes therapy has emerged as one of the most promising approaches in recent years for the treatment of diseases such as cancer and tissue repair. Exosomes extracted from crab haemolymph exhibit antioxidant activity and possess the ability to inhibit the proliferation of mouse breast cancer cells. Consequently, they are being considered as a potential treatment for breast cancer. However, the standalone use of exosomes presents limitations, including low bioavailability and a lack of controlled release mechanisms. To address these issues, the incorporation of extracellular vesicles into suitable biomaterial scaffolds has been proposed. This composite approach holds great potential in improving therapeutic outcomes and surpasses stem cells in terms of wound healing, bone regeneration, and cardiac repair (Khazaei et al. 2023; Rezakhani et al. 2021).

Bibliometrics is a broad discipline that integrates mathematics, statistics, and philology to quantitatively analyze the external structural features of knowledge vehicles (Smith 2008; Chen 2004; Moller and Myles 2016; Wallin 2005) and is now increasingly showing a trend to integrate life sciences with life medicine (Ma et al. 2020). CiteSpace is an information visualization software based on a scientific knowledge map from measurement and analysis of literature data developed by Professor Chen Chaomei, a Chinese scholar at Dreiser University in the United States (Chen 2004; Liu et al. 2014). Viewer is a document measurement visualization software developed independently by CWTS of Leiden University in the Netherlands (Eck and Waltman 2010; Eck and Waltman 2017). Both enable co-citation and clustering analysis of authors, journals, institutions, and keywords. However, due to the different data standardization algorithms and visualization presentation methods used by the two software, CiteSpace has advantages in revealing disciplinary dynamic development patterns and discovering research hotspots based on time series (Chen 2004). The VOSviewer software is preferred when the amount of node data is large or when the clarity of co-occurrence data is required (Chen and Song 2019).

So far, no comprehensive assessment or literature has been published on the relationship between exosomes and tumor microenvironments. Therefore, this study combined the two-visualization software mentioned above to summarize the global research activities on exosomes and tumor microenvironment from various aspects of bibliometric metrics. In addition, this study covers not only the latest developments in this field but cutting-edge research hotspots and future research advances and models. evaluation of research results in the field predicts future pathways and models and provides new directions for clinical diagnosis and treatment of tumors.

## Materials and methods

### Data sources and search strategies

We selected the Scopus database (http://www.scopus.com) for source publications retrieval as the target. Scopus is widely regarded as one of the best online databases for bibliometric research and is the largest abstract and citation database worldwide. Compared to other single abstract indexing databases, Scopus is more comprehensive and has a wider range of disciplines (Falagas et al. 2008; Sweileh 2020).

### Search strategies

We used the “advanced search” function of the Scopus online database and inserted appropriate keywords to find relevant literature on exosomes and tumor microenvironments for the databases created before May 4, 2022. To prevent bias caused by the ongoing database update, document extraction and export should be completed within 1 day (May 4, 2022). Terms related to exosomes and tumor microenvironment were extracted from PubMed's Medical Subject Headings (MeSH) in the Scopus engine. To make the results more comprehensive and precise, we used the search strategy of [MeSH] + free terms (Entry Terms), which are synonyms and polysemous terms to MESH. The search formula was as follows: (exosomes OR exosome) AND TITLE-ABS-KEY (cancer AND stem AND cells OR tumor AND stem AND cells OR neoplastic AND stem AND cells OR tumor AND microenvironment OR microenvironment, AND tumor OR microenvironments, AND tumor OR tumor, AND microenvironments OR cancer AND microenvironment OR cancer, AND microenvironments OR microenvironment, AND cancer OR microenvironments, AND cancer).

### Inclusion and exclusion criteria

Journal articles with research content related to the topic “Exosomes or exosome tumor stem cells and tumor microenvironment” were included by reading the titles, abstracts, and keywords of the detected articles. Articles with incomplete research information, conference articles, dissertations, book content, and duplicate articles were excluded. A total of 2077 publications were eventually included in the analysis, the basic information about which, including titles, abstracts, authors, affiliations, keywords, and references, were recorded on May 4, 2022.

### Bibliometric analysis

An Excel spreadsheet was used to collect the following data as bibliometric indicators: total number of publications, year of publication, the distribution of authors, the distribution of countries/regions, the distribution of journals, and the distribution of top‑cited publications.

### Visualized analysis

**Fig. 1:**
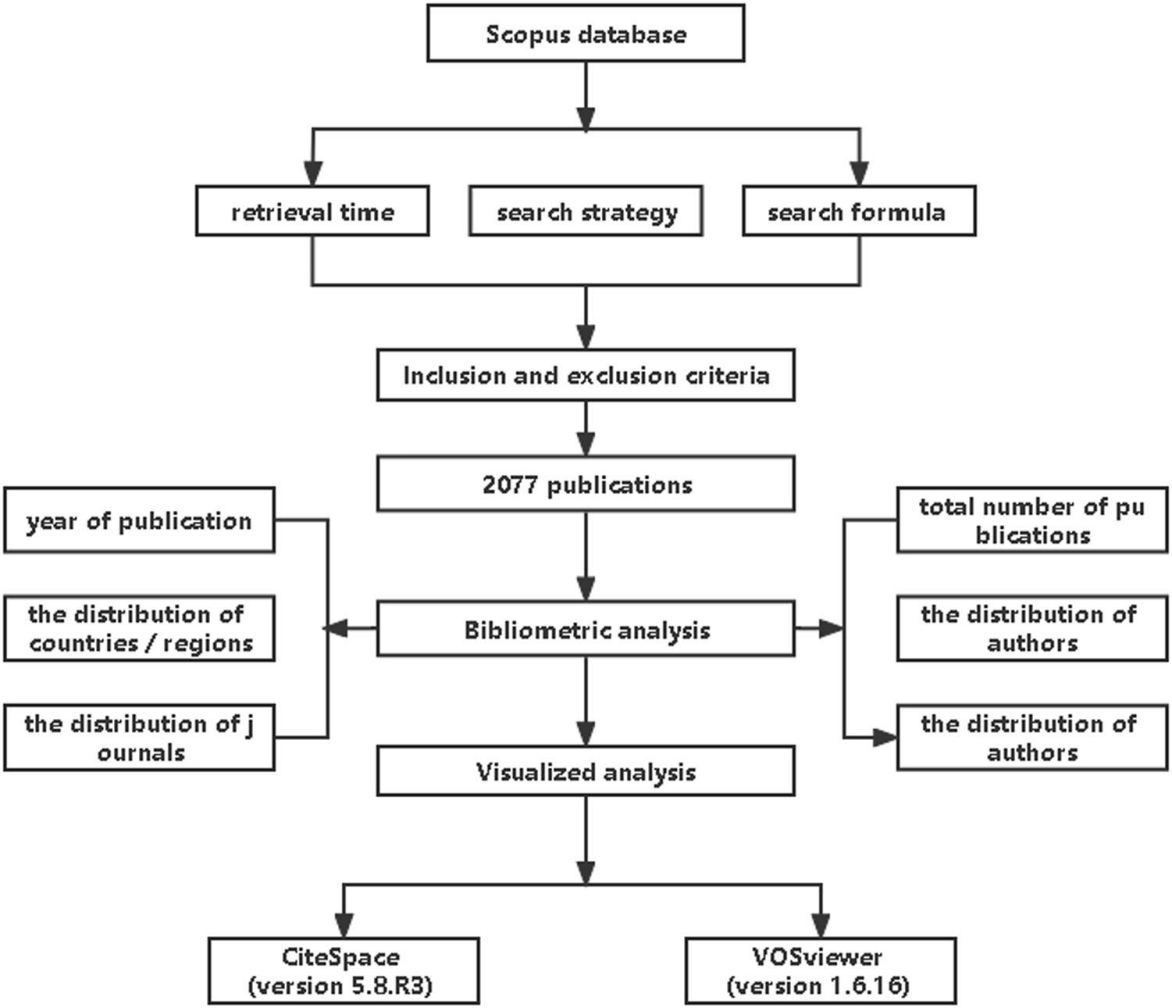
Methodological workflow for this study

CiteSpace (version 5.8.R3) and VOSviewer (version 1.6.16) software tools were used to build, visualize and explore networks of countries, authors, and terms, as well as links between co-occurrence-based items, and to create network visualization maps of the most co-occurrence terms to identify the hotspots for frontier studies, as well as the most co-authorship of countries. Methodological workflow for this study as show in Fig. 1.

## Results

### The trend of publication outputs

**Fig. 2:**
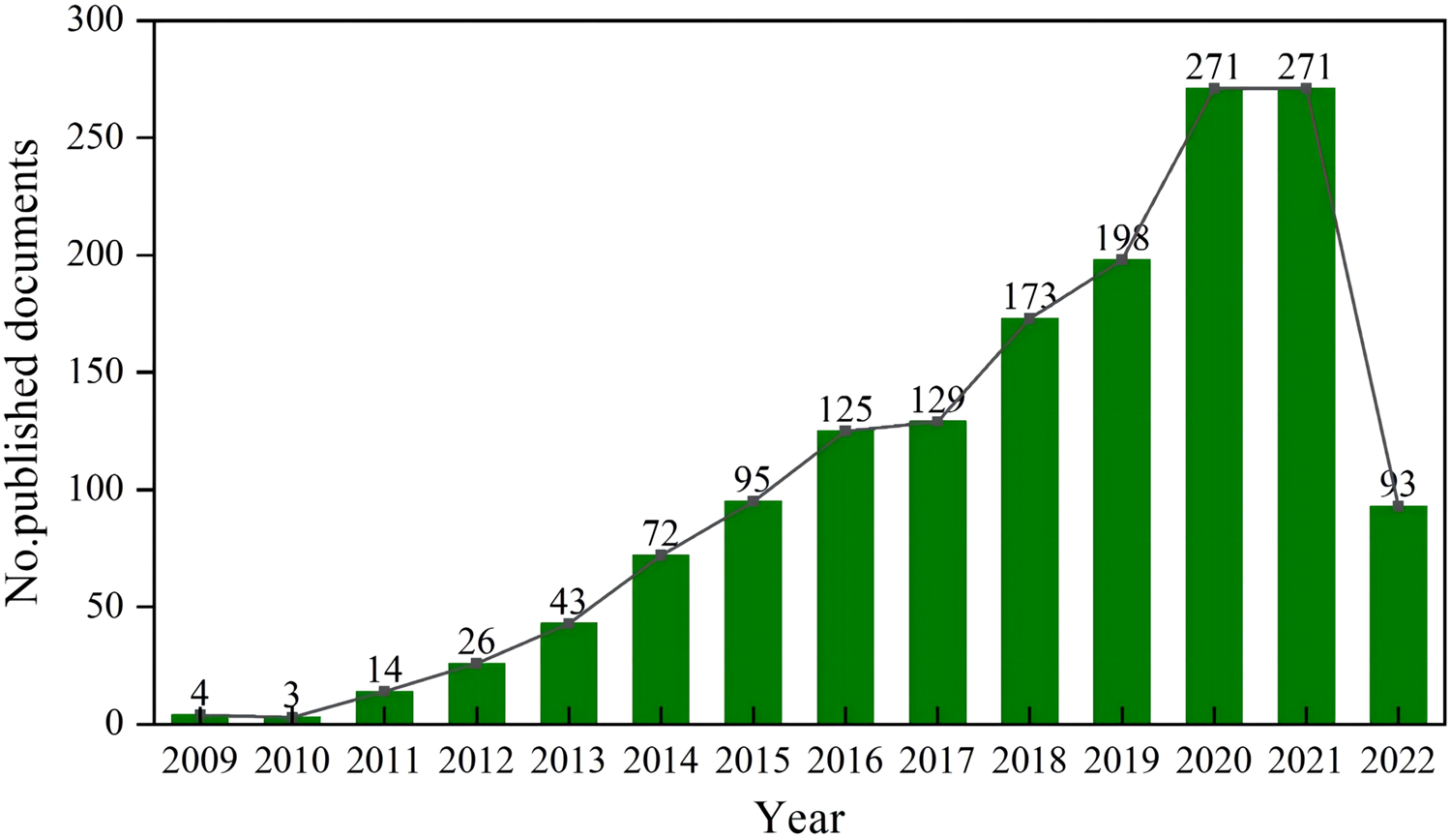
Trend of publication outputs from 2009 to 2022

The total number of publications focusing on the relationship between exosomes and tumor microenvironment from 2009 to May 4, 2022, was 2077, increasing from 4 in 2009 to 271 in 2021. The overall trend is increasing yearly, as shown in Fig. 2. The growth in the number of publications of related studies was divided into two phases. The first phase (2009–2016) grew gently, while the second phase (2017–2021) went more rapidly, reaching a peak of 271 and declining sharply to 93 in 2022.

### Distribution of authors

**Fig. 3:**
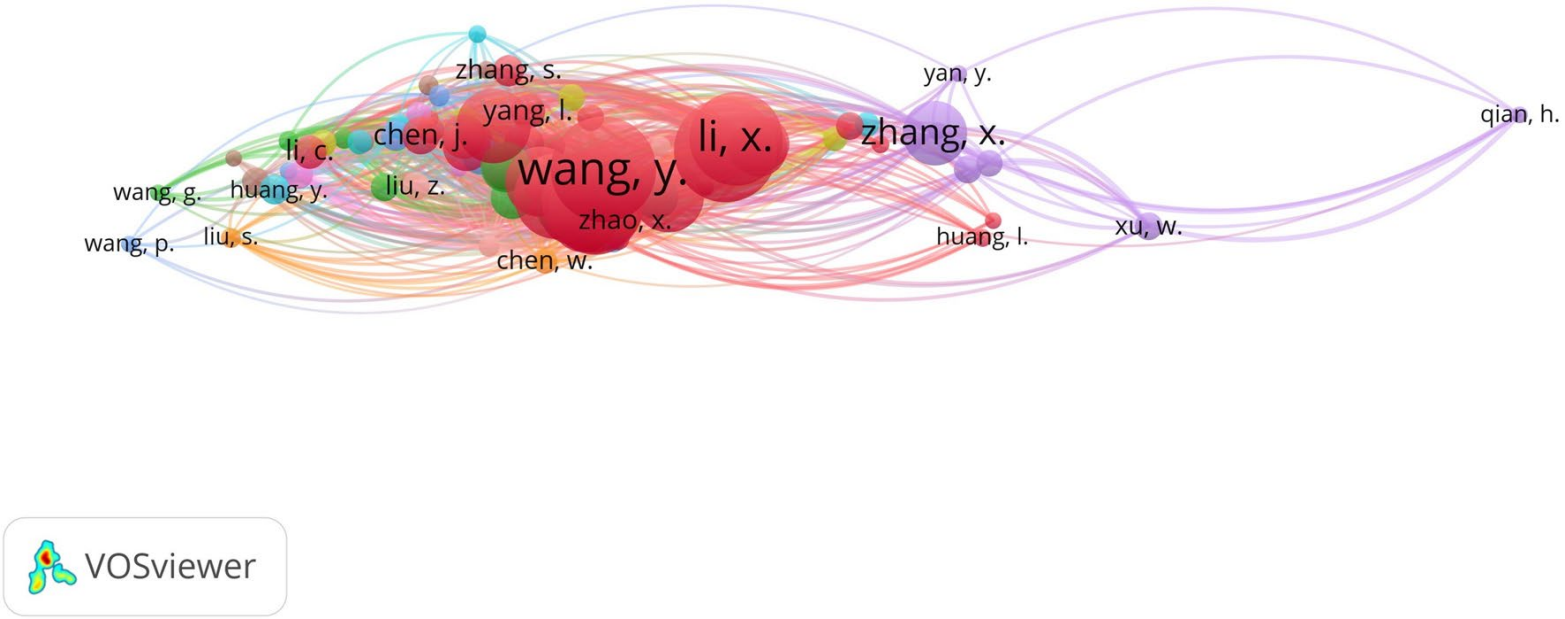
Distribution of authors

The co-occurrence analysis of the author collaboration network was performed by VOSviewer, as shown in Fig. 3. VOSviewer can be used to view the knowledge graph of the analysed domain in four views. In this study, we mainly use the labels view, which uses a circle and a label to represent an element, with the size of the circle representing the level of importance. Circles with the same colour belong to the same cluster. In order to avoid overlapping labels, the label view generally shows only a subset of labels, and the labels of each node on the graph can be viewed in detail by zooming in the software. Table 1 shows the top ten authors in terms of publication frequency, and the author with the highest number of publications is Wang, Y. The authors and their teams who published the most research in this field focused on the production and secretion of exosomes from tumours and tumour stem cells and their impact on cancer progression, emphasizing that exosomes secreted by tumour stem cells are regulated through the tumour stem cell-tumour microenvironment-target tissue interface, which can act as metastatic markers and are expected to serve as an entry point for blocking tumour metastasis. The author with the highest number of collaborative studies with other authors is ZHANG, Y.

**Table 1:** Top ten authors related to exosome tumor stem cells and tumor microenvironment

No	Frequency	Documents	Total link strength
1	WANG, Y	63	174
2	WANG, X	62	173
3	ZHANG, Y	62	193
4	LI, X	56	165
5	LIU, Y	56	136
6	WANG, J	51	147
7	ZHANG, J	45	110
8	LI, Y	43	115
9	LI, J	42	112
10	WANG, H	40	117

### Distribution of countries/regions

**Fig. 4:**
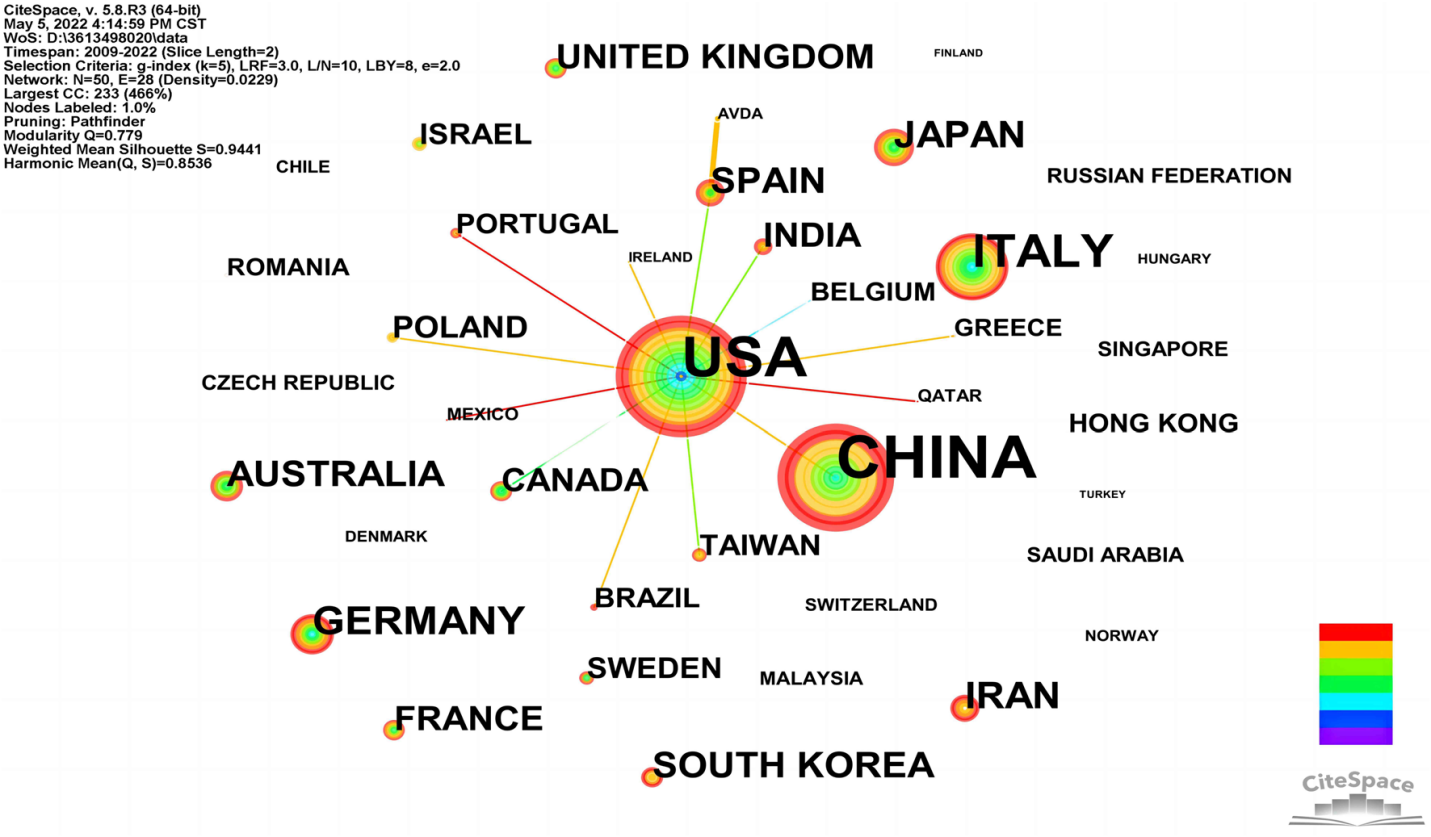
Distribution of publications from different countries/regions

The network mapping of countries/regions was created by CiteSpace visualization software, as shown in Fig. 4. Parameter settings for CiteSpace software to plot this figure: Slice Length = 2, Selection Criteria: g-index = (*k* = 5), LRF = 3.0, *e* = 2.0. The top five countries are China, the USA, Italy, Germany, and Japan, as shown in Table 2. Among them, China topped the list with 690 publications, accounting for about 33.22% of the total (2077). Centrality is an index to measure the importance of nodes in the network (Chen 2006) which is mainly used to measure the bridge function value of nodes in the whole network structure. Usually, nodes greater than 0.1 are considered relatively important. Majority of the EV research have been done in USA, China, Italy and many other researchers (top ten countries in Table 2). In addition, according to the results of the studies we included, the country with the least amount of relevant research in this area was Argentina, with a frequency of 2 and a centrality of 0.

**Table 2:** Top ten countries/regions related to exosome tumor stem cells and tumor microenvironment

No	Country	Frequency	Centrality	Year
1	CHINA	690	0.04	2012
2	USA	544	0.55	2009
3	ITALY	145	0.16	2009
4	GERMANY	61	0	2011
5	JAPAN	59	0	2013
6	IRAN	54	0	2015
7	SOUTH KOREA	46	0	2013
8	AUSTRALIA	44	0	2013
9	SPAIN	42	0.04	2013
10	FRANCE	38	0	2012

### Distribution of published journals

We performed a visual analysis of published journals using the VOSviewer software and found that a total of 572 academic journals published 2077 articles on exosomes and tumor microenvironment. Among them, *International Journal of Molecular Sciences* had the highest output with an impact factor (IF) of 5.923, followed by *Frontiers In oncology* with an IF of 6.244. Among the top ten academic journals, *Molecular Cancer* has the highest IF of 27.401. Specially, *Oncotarget* ranked fifth, reflecting the impact factor of 2017; however, the journal has not been included in Science Citation Index (SCI) since 2018. According to the Journal Citation Reports (JCR), 70% of journals belong to Q2, as shown in Table 3. It is clear that the impact of journals depends on the number of times they are cited together, which reflects whether the journal has a significant influence in a specific research field.

**Table 3:** Top ten journals related to exosome tumor stem cells and tumor microenvironment.* IF, impact factor; JCR, Journal Citation Reports*

No	Journal	Frequency	IF (2021)	JCR
1	International Journal Of Molecular Sciences	96	5.923	Q2
2	Frontiers In Oncology	79	6.244	Q2
3	Cancers	63	6.639	Q2
4	Molecular Cancer	49	27.401	Q1
5	Oncotarget	49	5.168 (2017)	Q2
6	Cancer Letters	48	8.679	Q1
7	Cells	39	6.6	Q1
8	Frontiers In Immunology	38	7.561	Q2
9	Frontiers In Cell And Developmental Biology	31	6.684	Q2
10	Advances In Experimental Medicine And Biology	26	2.622	Q2

### Distribution of top‑cited publications

**Fig. 5:**
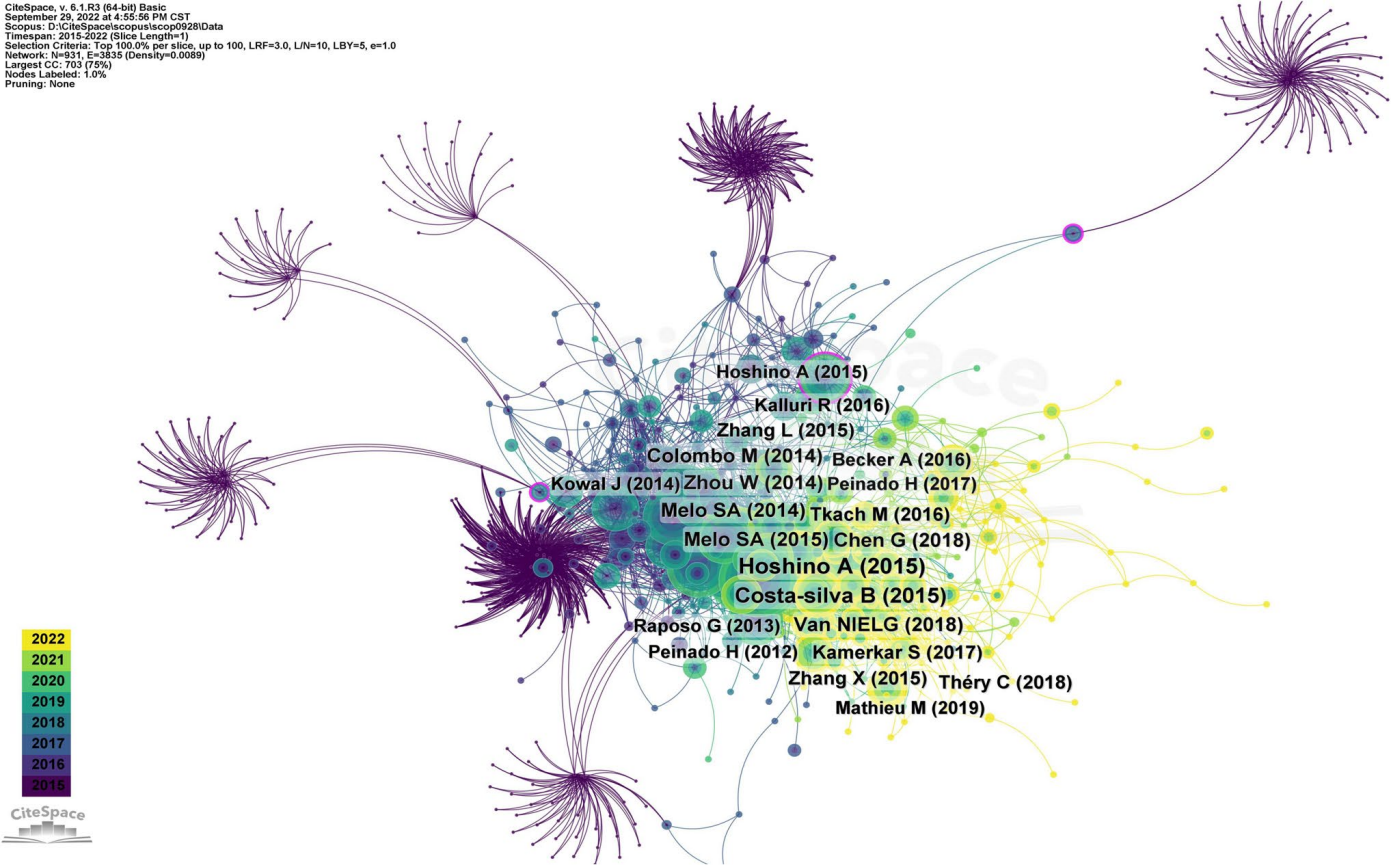
Distribution of top‑cited publications

We summarized the top ten most cited papers in the field of exosomal and tumor microenvironment since the establishment of the Scopus database, as shown in Table 4**.** Parameter settings for CiteSpace software to plot this figure: Slice Length = 1,Selection Criteria: LRF = 3.0, L/N = 10, LBY = 5, e = 1.0. The highest ten citations range from 161 to 65 (Hoshino et al. 2015; Costa-Silva et al. 2015; Niel et al. 2018; Chen et al. 2018; Melo et al. 2014; Melo et al. 2015; Zhou et al. 2014; Colombo et al. 2014b; Tkach and Théry 2016; Zhang et al. 2015). Hoshino A et al. (Hoshino et al. 2015), published in *Nature* in 2015, had the highest overall citation frequency (number of citations = 161). The publications co-citation network graph was created by CiteSpace visualization software, as shown in Fig. 5.

**Table 4:** List of the most frequently cited top ten highly cited papers related to exosomes tumor stem cells and tumor microenvironment

No	Centrality	Year	Authors	Source title	Cited by
1	0.04	2015	Hoshino A et al	Nature	161
2	0.04	2015	Costa-silva B et al	Nature cell biology	126
3	0.02	2018	Van NIELG et al	Nature reviews molecular cell Biology	89
4	0.04	2018	Chen G et al	Nature	85
5	0.01	2014	Melo SA et al	Cancer cell	82
6	0.01	2015	Melo SA et al	Nature	81
7	0.01	2014	Zhou W et al	Cancer cell	81
8	0.05	2014	Colombo M et al	Annual review of cell and Developmental biology	75
9	0.01	2016	Tkach M et al	Cell	69
10	0.02	2015	Zhang L et al	Nature	62

### Keywords visualization analysis

#### Keywords co-occurrence analysis

**Fig. 6:**
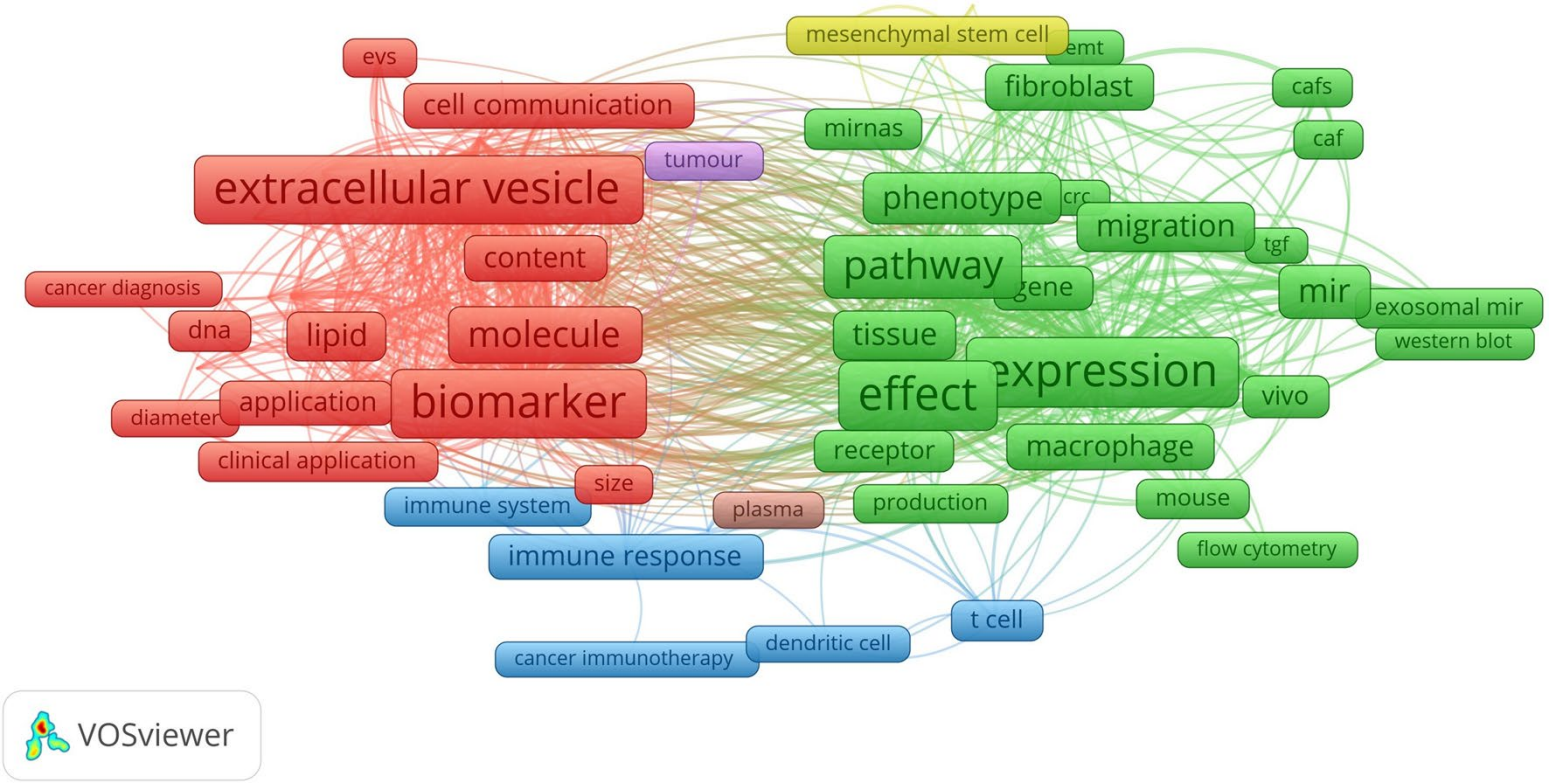
Network visualization map of terms in title/abstract fields of publications related to exosome tumor stem cells and tumor microenvironment

**Fig.7:**
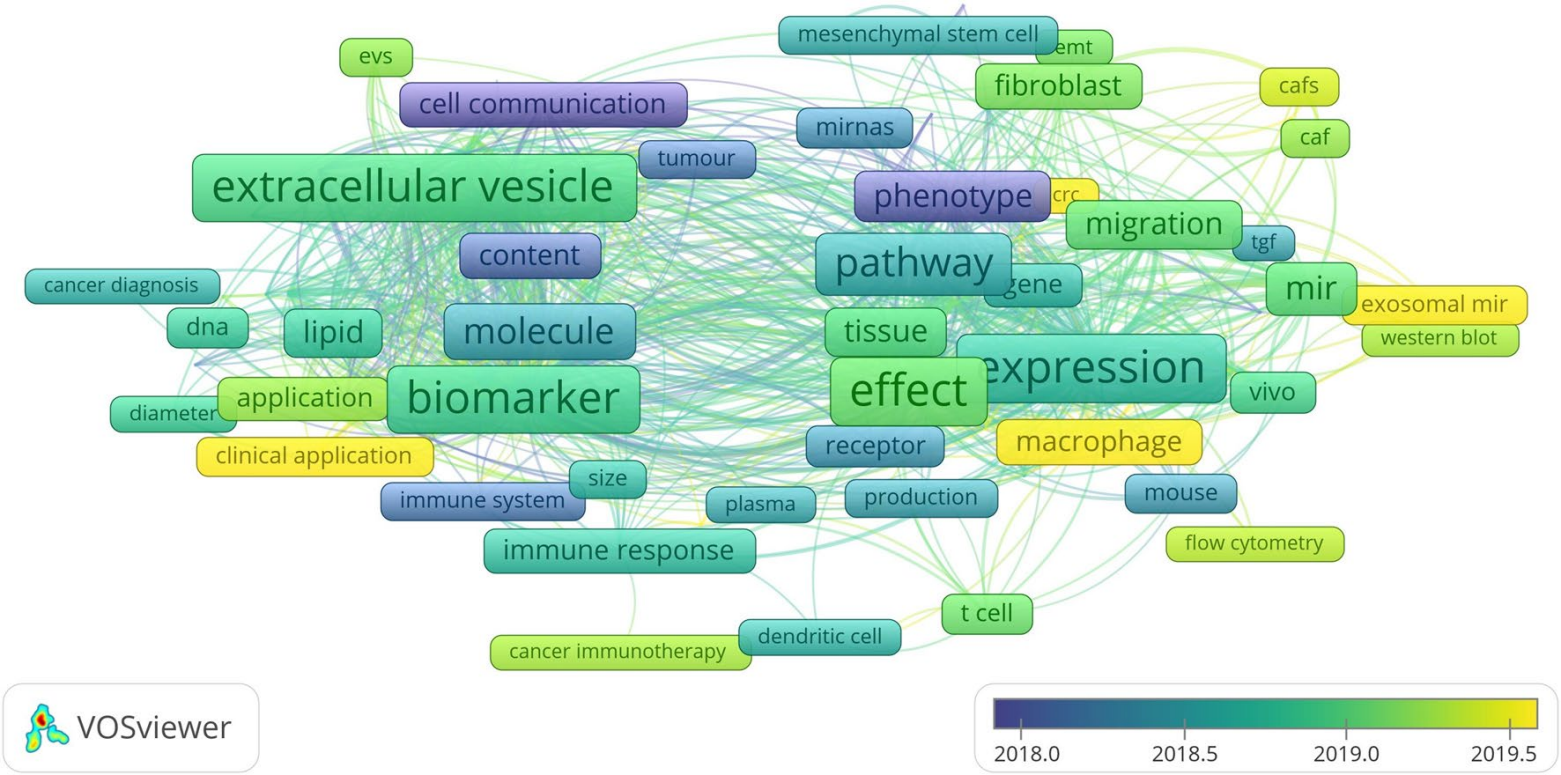
Network visualization map of terms in the title/abstract and their distribution according to the mean frequency of appearance. The blue terms appear first, followed by the yellow and green terms

Keywords are a high-level summary and overview of the core viewpoints and themes of the included literature, which can reflect the hotspots and frontiers of the field. Terms with at least > 50 occurrences in all included publications were selected for analysis using VOSviewer. Of the 31,085 terms in the field, 230 terms reached this threshold, and they were divided into three clusters with different colors, as shown in Fig. 6. Boxes and labels form an element, the size of the element depends on the degree of the node, the strength of the connectivity, the amount of citations, etc., and the colour of the element represents the cluster to which it belongs, with different clusters represented by different colours. The cluster of red boxes in this figure indicates that the keywords in that colour have high weight or frequency and become the core words of the cluster, followed by green and blue. In cluster 1(red), the most striking keywords are extracellular vesicle, biomarker, molecule, cell communication, and vesicle. In cluster 2 (green), the most frequent keywords are expression, effect, pathway, level, phenotype, and migration. In cluster 3 (blue), the most repeated keywords are immune response, immune system, cancer treatment, dendritic cell, and t cell. These results demonstrated that over the past decade, the research on the relationship between exosomes and cancer microenvironment has focused on three main areas: “research on tumor biomarkers represented by exosome nucleic acids and proteins”, “research on the mechanism represented by tumor proliferation, metastasis, growth, and apoptosis”, “research on tumor immunity around immune cells and cytokines.” the VOS viewer color-coded the keywords based on their average time of appearance in 2077 relevant publications, as shown in Fig. 7. The terms in blue appeared earlier, and those in yellow and green were later. Until 2019, most studies focused on the “research on the mechanism represented by tumor proliferation, metastasis, growth, and apoptosis.” Recent trends suggest that exosome as cell-to-cell messaging carriers will receive more attention in the search for exonic tumor markers and their application in tumor immunotherapy.

#### Keywords cluster analysis

**Fig. 8:**
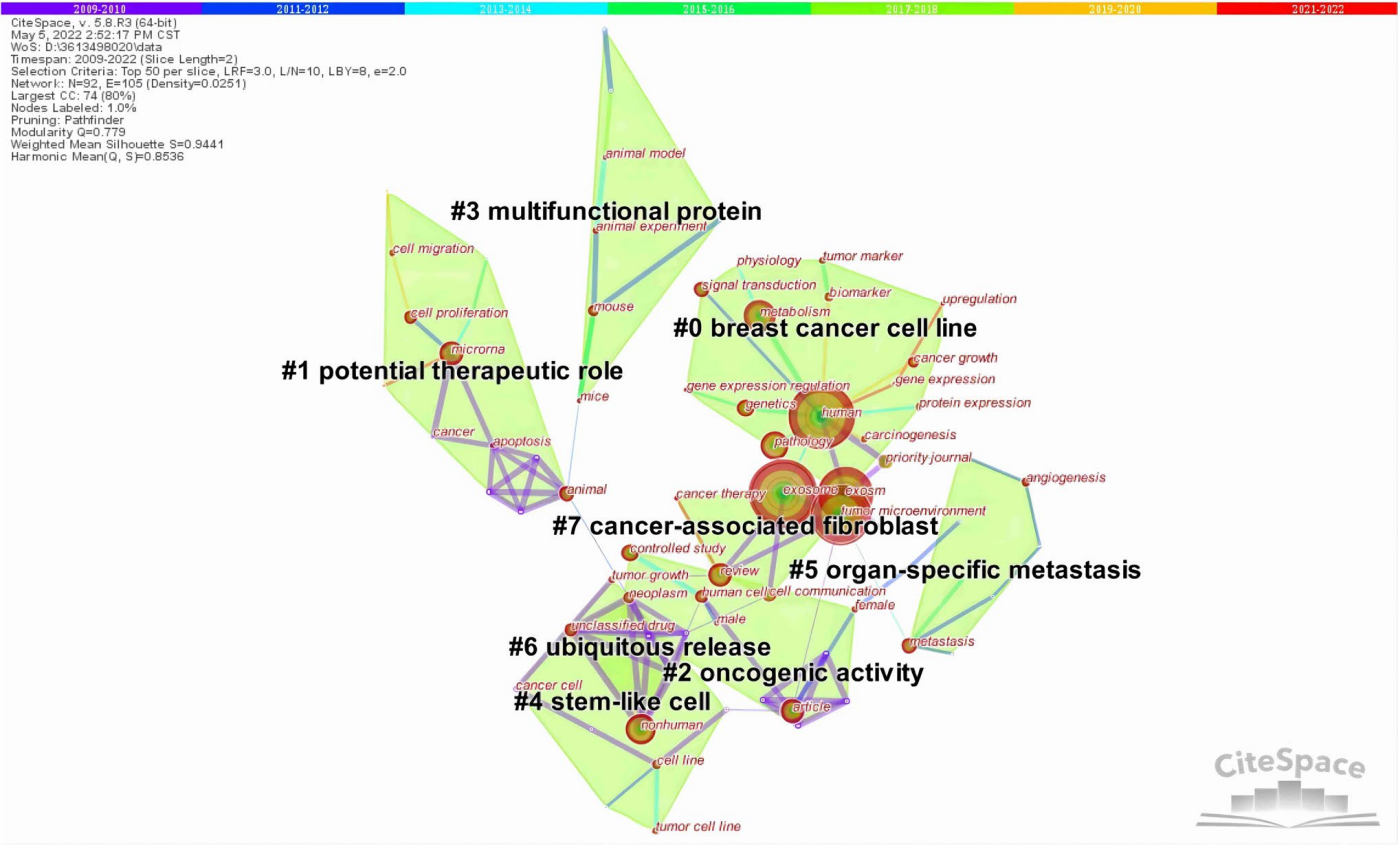
CiteSpace visualization map of co-occurring keywords clusters

Each cluster is composed of multiple closely connected keywords. The smaller the number of cluster IDs on the map, the more keywords are included. The clustering Modularity (*Q* value) and the Mean Silhouette (*S* value) can be used as a basis for evaluating effectiveness of the mapping. In general, *Q* > 0.3 means that the community structure is distinct. Clustering with S > 0.5 is generally considered reasonable. When *S* value is 0.7, the clustering is convincing. Based on the keywords co-occurrence analysis mapping, the clustering of the relationship between exosomes and tumor microenvironment using CiteSpace resulted in eight research directions related to them, as shown in Fig. 8, including breast cancer cell line, potential therapeutic role, cancer-associated fibroblast, etc. Clustering identifies the knowledge structure foundation and dynamic evolution process of this research field: for instance, cluster 5, “organ-specific metastasis,” contains early neoplastic lesion, targeting cells, metastatic dormancy, and liver metastasis. Cluster 7, “cancer-associated fibroblast,” included tumor-associated macrophages, prostate cancer, tumor growth, and exosomal transfer. The above clustering results also suggest that exosome derived from tumor cells can be released extracellularly as carriers of intercellular information transfer and play an important role in promoting tumor invasion and metastasis.

#### Keywords burst analysis

**Fig. 9:**
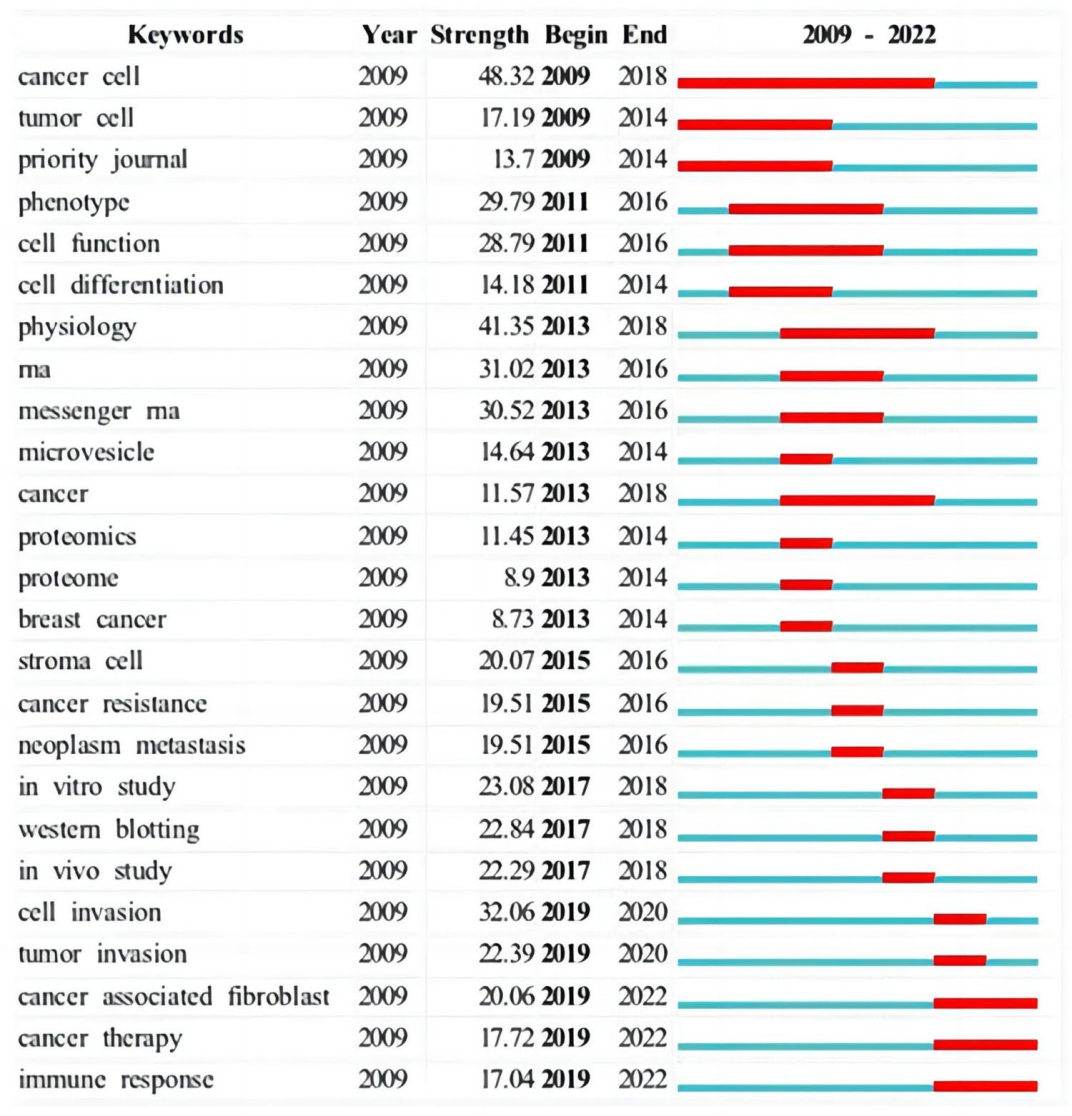
CiteSpace visualization map of top 25 keywords with the strongest citation bursts related to exosome tumor stem cells and tumor microenvironment

Burst words detect high-frequency words by the change of keyword frequency in a specific time period, which to a certain extent reflects the hot spots of burst research directions in the field, i.e., research frontiers or hot spots. The top 25 burst words in the field of exosome and tumor microenvironment research are shown in Fig. 9. Cancer cell (48.32) and physiology (41.35) were the most frequently changed keywords, indicating that these two sectors are an important frontier. In 2019, research mainly focused on cell invasion, tumor invasion, cancer associated fibroblasts, cancer treatment, and immune response. It continued to present, which further indicated that exosomes play a key role in mediating intercellular information exchange, tumor invasion, and metastasis. As a drug carrier, exosomes have gradually become the mainstream in tumor immune regulation and treatment research, and the research content is also gradually deepening. It is expected to continue to be a research hotspot and explore potential and valuable research directions.

#### Keywords co-occurrence time zone map analysis

**Fig. 10:**
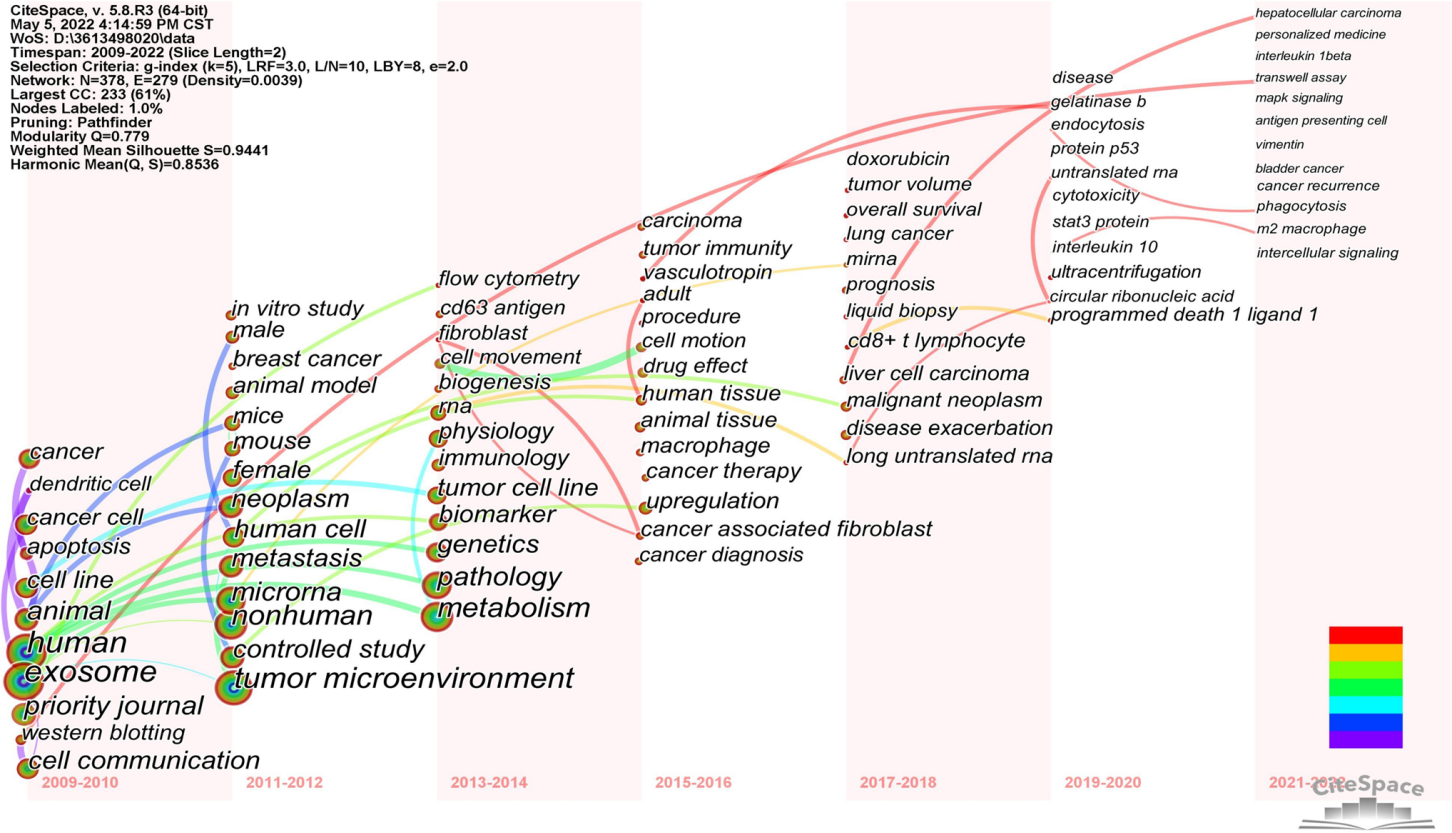
CiteSpace visualization map of co-occurrence time zone related to exosome tumor stem cells and tumor microenvironment

The co-occurrence time zone map helps better demonstrate the research hotspots and temporal evolution trends of exosomes and tumor microenvironment, and comprehensively analyze the changing patterns of major research themes in this field over the time span. Keywords were analyzed according to years of 2009–2022, divided into 2-year time zones, and the keyword co-occurrence time zone map was drawn (Fig. 10), which to a certain extent indicates the stage-specific characteristics of relevant research in this field. Parameter settings for CiteSpace software to plot this figure: Slice Length = 2, Selection Criteria: g-index = (k = 5), LRF = 3.0, L/N = 10, LBY = 8, e = 2.0. As far as the position is concerned, from 2009 to 2012, the main research directions were quite basic, through the construction of animal models and design of animal experiments, to reveal the principle and mechanism of exosomes as intercellular communication carriers. Breast cancer was one of the first tumors studied, and several studies have now shown that exomes play an important role in breast cancer progression, migration, and drug resistance (Yang et al. 2022; Zuo et al. 2022). Since 2015, the main research directions in this field included two aspects: First, the discovery of an increasing number of tumor markers in exomes, which have an early diagnosis of tumors with certain guidance significance. Research on related diseases includes lung cancer, hepatocellular carcinoma, prostate cancer, bladder cancer and so on. Second, in terms of tumor molecular mechanism, for example, exosomes can not only directly regulate the growth of cancer cells, but also affect the activation of immune cells and the secretion of cytokines, which enable tumor cells to avoid immune attack. Moreover, non-coding RNAs and growth factors in tumor exosomes can promote tumor growth and metastasis by altering the tumor microenvironment.

## Discussion

### General information

This study visualizes the overall situation and research hotspots of exosomes and tumor microenvironment-related studies and cases in the past decade through visual maps. A total of 2077 publications originating from the Scopus database were analyzed. According to several studies, Scopus is the world’s largest database of abstracts and citations from peer-reviewed scientific literature (Falagas et al. 2008; Kulkarni et al. 2009), which is a multidisciplinary database with more indexed journals than PubMed and WoS. CiteSpace and VOSviewer have been widely used visualization software with multiplicity, dynamism, and time-sharing that can present the development status and trends of a research field and help researchers grasp the hot spots and frontiers of scientific research.

The number and trend of publications per year can reflect the development rate and research progress of the study and indicate the concentration of research in the field. From 2009 to May 4, 2022, the number of published articles on the relationship between exosomes and tumor microenvironment has been increasing yearly. The growth in the number of publications of related studies was divided into two phases. The second phase (2017–2021) increased more quickly than the first phase (2009–2016), which developed more slowly. In the first stage, the growth rate is relatively slow. The primary explanation may be that as the area of miRNA and tumor microenvironment research continues to advance, researchers' focus is shifting inexorably toward the control of exosomes produced by tumor cells and miRNAs in the tumor microenvironment. The primary explanation for why the second stage of development occurs so quickly is that exosomes have a variety of immune regulatory functions that may both encourage and inhibit immunological response, according to an increasing number of studies (Zuo et al. 2022). Second, exosomes can change the sensitivity of cancer cells to chemotherapy drugs by regulating cell signaling pathways. Finally, exosomes have gradually attracted attention in tumor diagnosis and prognostic biomarkers, and the research results have provided new ideas for researchers. In addition, according to the results of the study, the related studies of exosomes and tumour microenvironment showed a decreasing trend in 2022. We considered that it might be related to the time of our search, which was from the time of library construction to 4 May 2022, and the related studies in the second half of 2022 have not been counted, so the results have a certain lag.

From the perspective of national and regional distribution, China has the most significant number of publications, followed by the United States, demonstrating that American and Chinese academics are the primary research forces in this area. Among the top ten countries, the United States has the highest centrality (0.55), meaning it plays a key role in the global network of national cooperation, followed by Italy (0.16) and China (0.04). The diversity of the researchers with this knowledge and the sizeable financial support for researchers are two factors that contribute to the success of research in these nations. Given that the nations involved have better developed infrastructures, greater availability of scientific services, and a long tradition in the general study of exosomes and the tumor microenvironment, the research output is not surprising. However, there is still a dearth of deep cooperation and information sharing, and country research is still mostly independent. Therefore, strengthening cooperation and exchange among research institutions and researchers in different countries is beneficial to the flow of information, innovation of research methods and breakthrough of current research bottlenecks.

We discovered from the distribution of journals that 572 academic publications published 2077 papers about exosomes and the tumor microenvironment. The journals with the most articles about exosomes and tumor microenvironment were *International Journal of Molecular Sciences*, followed by *Frontiers In Oncology* and *Cancers*. Among the top ten academic journals, *Molecular Cancer* has the highest impact factor of 27.401. Finding the key journals that published research on exosomes and the cancer microenvironment is made easier by analyzing the distribution of literature sources. From the distribution of top‑cited publications, we summarized the top ten most cited papers in the field of exosomal and tumor microenvironment since the Scopus database was built; the highest ten citations range from 161 to 165. Among the top ten publications with the highest total citation frequency, Hoshino A et al. published in *Nature* in 2015 had the highest total citation frequency (number of citations = 161).

### The hotspots and frontiers

Keywords are the research themes and core contents of the literature. Using term co-occurrence analysis, it is feasible to comprehend the distribution and growth of various research hotspots in a specific topic. It can be seen in Fig. 6. Keywords with high frequency are extracellular vesicles, biomarker, molecule, cell communication, expression, effect, pathway, phenotype, migration, immune response, immune system, cancer treatment, etc. Based on keywords co-occurrence analysis map, cluster analysis, burst word analysis and time zone diagram analysis of the relationship between exosomes and tumor microenvironment were mapped by CiteSpace, and the research hotspots and development frontiers in this field were further determined.

CSCs have the capacity to reverse differentiation, self-replenishment, and self-renewal in tumor tissues, and have potential migration and drug resistance characteristics. They are regarded as cancer-causing agents because they facilitate the development, spread, and recurrence of cancer as well as treatment resistance (Clara et al. 2020; Ayob and Ramasamy 2018). Exosomes produced by tumor stem cells have been shown to be essential for tumor development and progression in recent years due to their ability to control TME elements locally or remotely in an autocrine or paracrine manner (Peitzsch et al. 2017). According to our study, the connection between CSC-Exos and TME is mainly reflected in the fact that exosomes regulate the growth and metastasis of tumor cells, participate in tumor angiogenesis and have potential value as tumor diagnostics (Tan et al. 2020; Zhao et al. 2022; He et al. 2019; Zeng et al. 2018). In addition, it has also been shown that because CSC-Exos deliver key molecules that are responsive to chemotherapy and immunotherapy, they may also contribute to tumor drug resistance and impede effective responses to antitumor immunotherapy, leading to poor clinical outcomes (López de Andrés et al. 2020; Han et al. 2020). Therefore, the targeting of CSC-Exos as therapeutic agents and drug delivery vehicles to inhibit or remove cancer cells may offer promise for future applications in cancer therapy (Liu et al. 2022a; Aramini, et al. 2022).

Epithelial–mesenchymal transition (EMT) is a critical step in cancer metastasis and infiltration. CSC-Exos acts as a transporter of EMT initiation signals and delivers these signals to tumor cells, leading to cancer metastasis and infiltration (Jiang et al. 2022). In addition, angiogenesis refers to the ability of an organism to form a new vascular system based on the primitive vascular system. Tumor growth requires blood vessels to provide various nutrients and is a key step in tumor development (Du et al. 2020). Exosomes can regulate intercellular communication through proteins and RNA, leading to alterations in tumor heterogeneity and ultimately promoting malignant proliferation and an aggressive cellular phenotype of tumor cells (Zhao et al. 2022). Or exosomes regulate vascular permeability by increasing the level of angiogenic factors in TME. Hepatocellular carcinoma (HCC) is a highly angiogenic cancer. Lin found that miR-210 secreted by HCC cells can promote tumor angiogenesis by targeting SMAD4 and STAT6 to endothelial cells (Lin et al. 2018). Sarcoma delivers miRNA to endothelial cells via exosomes to increase the expression of angiogenic factors including VEGF-A, IL-6 and IL-8 (Raimondi et al. 2020). Due to advances in liquid biopsy technology for early cancer detection, exosomes may become an important tool for early cancer diagnosis and prognosis (Kok and Yu 2020; Kumar et al. 2015). Circulating exosomal circRNAs and exosomal proteins have also been shown to reflect the progressive and malignant features of cancer, and they have great potential as non-invasive biomarkers for cancer diagnosis and prognosis (Wang et al. 2020; Seimiya et al. 2020; Li et al. 2017). Pan (Pan et al. 2018) found eight miRNAs in the plasma exosomes of ovarian cancer patients compared with healthy women. Among them, four miRNAs (miR-21, miR-100, miR-200 b, and miR-320) were significantly enriched in the plasma exosomes of ovarian cancer patients, while the other four miRNAs (miR-16, miR-93, miR-126 and miR-223) were insufficiently expressed in the exosomes of ovarian cancer patients. This research reveals that exosome metastasis is a mechanism through which miRNAs produced from ovarian cancer influence the local and distant environment. They are not only involved in tumor growth, angiogenesis, invasion, metastasis, and immunosuppression but also can be used as a new biomarker for early diagnosis of ovarian cancer. Yang (Yang et al. 2020) found that the plasma exosomes of colorectal cancer patients were rich in circ133, and the expression of circ133 increased with the progression of the disease and was related to cell hypoxia. Hypoxia cell exosome circ133 promotes tumor cell metastasis by targeting the miR-133a/GEF-H1/RhoA signaling pathway, and exosome circ133 is expected to become a biomarker for monitoring the progression of colorectal cancer. Based on the evidence of these research, the combination of multiple components involved in exosomes may help enhance the specificity and sensitivity of cancer diagnosis, while further research is still needed.

They are natural nanocarriers secreted by various cells, making them suitable candidates for diverse drug delivery and therapeutic applications from a material standpoint. They have a phospholipid bilayer decorated with functional molecules and an enclosed parental matrix, which has attracted interest in developing designer/hybrid engineered exosome nanocarriers. The structural versatility of exosomes allows the modification of their original configuration using various methods, including genetic engineering, chemical procedures, physical techniques, and microfluidic technology, to load exosomes with additional cargo for expanded biomedical applications. Thus, such research has great potential for future application in personal medicine (Mondal et al. 2023; Li et al. 2019).In addition, chemotherapy, as a frontline treatment option for cancer, is associated with limitations such as low tumor penetration efficiency, low bioavailability, local toxicity, and poor solubility in fluids. To address these challenges, utilizing the delivery properties of exosomes to load chemotherapy drugs has emerged as a potential solution. This cancer-specific drug delivery technology holds the potential to improve patient survival rates and greatly reduce the need for high-dose drug injections. Currently, researchers have been exploring the application of exosomes in drugs such as doxorubicin, paclitaxel, and cabazitaxel (Rezakhani et al. 2022). Moreover, exosomes have emerged as the most promising miRNA carriers due to their high permeability, long half-life across biological barriers, and natural ability to transport cargo as shuttle carriers under physiological and pathological conditions (Boorn et al. 2013). However, exosomes are cell heterogeneous and differences in their size give them different contents and functions as well as limited drug delivery efficiency, raising questions regarding their safety (Chen et al. 2022; Nicolini et al. 2021). Therefore, the development and clinical application of exosomes as drug delivery systems remains challenging and deserves further research and investigation.

In recent years, SPR (Surface Plasmon Resonance technology, SPR), AFM (Atomic Force Microscopy, AFM), and microfluidic device, as advanced biosensing tools, have been increasingly used to analyse the binding specificity between biomolecules. For example, SPR technology can be used for real-time detection of interactions between DNA and proteins, protein molecules, drugs and proteins, nucleic acids and nucleic acids, antigens and antibodies, and receptors and ligands, among other biomolecules. AFM, is an analytical instrument that can be used to study the surface structure of solid materials, including insulators. It investigates the surface structure and properties of substances by detecting the very weak interatomic interaction forces between the surface of the sample to be examined and a miniature force-sensitive element (i.e. the probe). Microfluidic systems, on the other hand, are used in precision medicine, and functional microfluidic assays as therapeutic predictors are promising and valuable for exploring the relationship between exosomes and the tumour microenvironment (Ayuso et al. 2022).

## Limitations

CiteSpace and VOSviewer cannot completely replace system retrieval, and there are still some limitations to be addressed. First of all, the literature we obtained was from 2009 to 4 May 2022. The retrieval findings of this study, however, deviate significantly from the actual number of included literature due to the ongoing updating of the literature in Scopus. Second, this study includes both articles and reviews, the gathered literature's variable quality might undermine the validity of the map analysis. Finally, because several important keywords from the manuscript were only partially extracted throughout the analysis, the outcomes could have been impacted. Nevertheless, the visualized analysis based on literature undoubtedly lays a foundation for scholars to quickly understand the research subjects, research hotspots and development trends in the field of exosomes and tumor microenvironment.

## Conclusions

This study shows the current status and hot directions of tumour stem cell-derived exosomes and tumour microenvironment worldwide. Research on the mechanisms of immune response, immune escape, immune tolerance, tumour invasion and metastasis of CSC-Exos in TME is still a hot topic. As a key intercellular communication tool, exosomes are increasingly being mined by researchers for their rich bioinformatic molecules, and their blossoming potential has gained great and continuous attention. In the future, there will be more and more research results on exosomes in the fields of early diagnosis of tumours, disease monitoring, evaluation of therapeutic response and drug delivery, which will provide new perspectives and strategies for early diagnosis of tumours and overall management of tumours.

## Data Availability

The original contributions presented in the study are included in the article. Further inquiries can be directed to the corresponding authors.

## Abbreviations

TME: The tumor microenvironment
CSC-Exos: Cancer stem cell-derived exosomes
miRNA: MicroRNA
BCSCs: Breast cancer stem cells
IF: Impact factor
JCR: Journal Citation Reports
EMT: Epithelial–mesenchymal transition
HCC: Hepatocellular carcinoma
